# Annealing Effect on the Structure and Optical Properties of CBD-ZnS Thin Films for Windscreen Coating

**DOI:** 10.3390/ma14226748

**Published:** 2021-11-09

**Authors:** Raghad Y. Mohammed

**Affiliations:** Department of Physics, College of Science, University of Duhok, Duhok 42001, Iraq; ssraghad@uod.ac

**Keywords:** ZnS, chemical bath deposition, annealing, energy gap

## Abstract

Zinc sulfide (ZnS) thin films were prepared and synthesized by the chemical bath deposition (CBD) technique on microscopic glass substrates using stoichiometric amounts of the precursor materials (ZnSO_4_·7H_2_O, NH_4_OH, and CS(NH_2_)_2_). Structural, morphological, compositional, and optical characterization of the films were studied. The obtained thin films were found to exhibit polycrystalline possessions. The effect of annealing temperature on the crystallographic structure and optical bandgap of ZnS thin films were both examined. The grain size and unit cell volume were both found to be increased. In addition, the strain, dislocation density, and the number of crystallites were found to be decreased with annealing temperature at 300 °C. However, the annealed sample was perceived to have more Zn content than S. The optical characterization reveals that the transmittance was around 76% of the as-deposited thin film and had been decreased to ~50% with the increasing of the annealing temperature. At the same time, the bandgap energy of the as-deposited film was 3.98 eV and was found to be decreased to 3.93 eV after annealing.

## 1. Introduction

ZnS is an II-VI compound semiconductor that is found in two forms: a cubic form (c-ZnS) and hexagonal form (h-ZnS). At normal conditions of temperature and pressure, it crystallites in a zinc-blended structure [1]. Recently ZnS has been studied extensively because of its wide applications in optoelectronic devices due to direct broad bandgap 3.6~3.8 eV in the bulk and considerable exciton binding energy [2,3,4]. The broad bandgap of ZnS makes it a very significant material for UV-LED [5], flat panel displays [6], windows for solar cells, and sensors [7].

ZnS thin films can be synthesized by several techniques such as Chemical Vapor Deposition (PVD) [8], spray pyrolysis [9], RF sputtering [10], Chemical Bath Deposition (CBD) [11], plasma-assisted MOCVD [12], and SILAR method [13]. Among them, CBD is well known as a simple, most economical, and low-temperature technique for depositing large-area thin films of semiconductors. During the CBD process, the substrate is immersed in a solution containing a chalcogenide source (X), a metal ion (M+), an added base, and a complexing agent. The process depends on the slow release of chalcogenide ions into an alkaline mixture where film formation on the substrate takes place when the ionic product (IP) exceeds the solubility product (SP) [14].

Large numbers of binary semiconductors compound like CdS [15,16,17], CdSe [18,19,20], PbS [21,22,23], CuS [24,25,26], and ZnS [27,28,29] can be deposited as thin films using CBD method. For the deposition of ZnS thin films via CBD, some factors should be considered and controlled. These factors include Zn precursor, S precursor, complexing agent, pH of the mixture, bath temperature, and deposition time [30,31,32,33,34,35].

In this paper, ZnS thin films are prepared by the chemical bath deposition technique. The prepared films were annealed at 100, 150, 200, 250, and 300 °C. The effect of annealing temperatures on the structure and bandgap of the prepared films are investigated.

## 2. Materials and Methods

**Figure 1:**
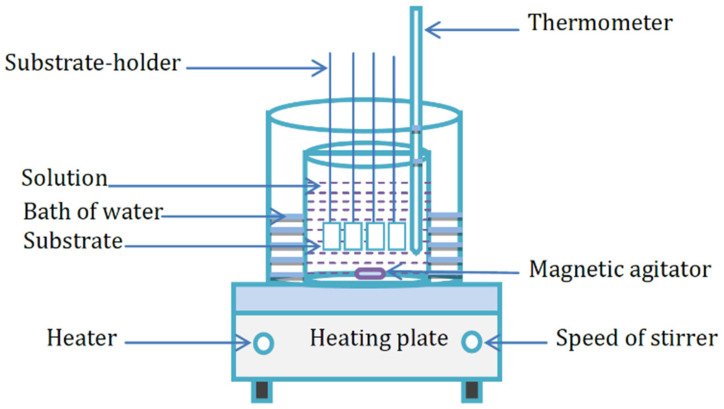
Chemical bath deposition system.

ZnS thin films were synthesized on microscopic glass substrates (75 mm × 25 mm × 1 mm). Figure 1 reveals the CBD setup used for this purpose.

The glass substrates were cleaned carefully. They were first dipped into a chromic acid and set aside for 24 h. Then, they were cleaned in distilled water with ultrasonic vibration for 15 min, immersed into acetone, rinsed again with double distilled water. Finally, they were dried with air and kept in a desiccator [16].

ZnS thin films were prepared by immersing the substrate in a beaker containing the mixture of precursor materials (0.03 M zinc sulfate heptahydrate (ZnSO_4_·7H_2_O) as Zn^2+^ ion source, 0.5 M thiourea as a source of S^2−^(CS(NH_2_)_2_), and 2.5 M of ammonia (NH_4_OH) as a complexing agent) at room temperature with continuous stirring and placed in a water bath (T_b_ ± 2). They were then heated to the needed deposition temperature of (60 °C). The pH was adjusted to be (9.8 ± 0.1) with continuous stirring. The substrates were then taken out after 45 min (deposition time) and washed with distilled water to remove the porous zinc-sulfide over the layer; then, they were left to be dried. Thin and uniform ZnS thin films have been obtained. An optical interferometer method [25,36] was used to calculate their thickness.

The deposited films were then being annealed at 100, 150, 200, 250, and 300 °C for 60 min by a tube type of furnace. The structural and optical properties were then examined to determine the effect of annealing temperature on these characteristics. X-ray diffraction (XRD) spectra were recorded with an XPERT-PRO(X-Pert Pro PANalytical) diffractometer using CuKα radiation (λ = 1.5406 Å, with 45 kV and 40 mA) for 2θ values over 20° to 80°. The composition, morphology of the surface, along with the optical properties of the films, have also been characterized by an Energy-dispersive X-ray Spectroscopy (EDX, FEI Company, US), Field-emission scanning electron microscopy (FESEM) (450 Quanta, FEI Company, US), and the UV–VIS Spectrophotometer (6850 UV/Vis. Spectrophotometer-JANEWAY, Cole-Parmer Ltd., Stone, Staffordshire, UK), respectively, in the wavelength range 190–1100 nm.

## 3. Results

### 3.1. Structure Analysis

**Figure 2:**
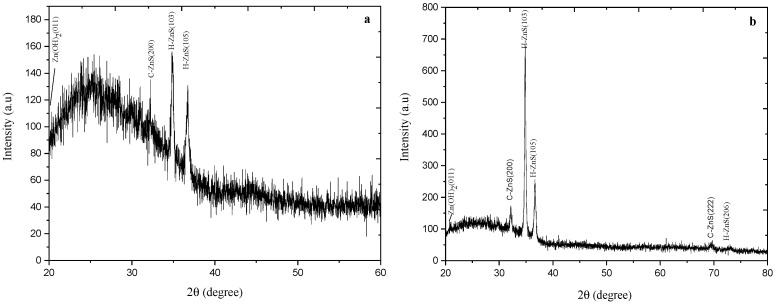
XRD pattern for as-deposited ZnS thin films (**a**), and (**b**) annealed ZnS thin films at 300 °C for one hour.

X-ray diffraction measurements have been carried out to study the structure of the as-deposited and annealed ZnS thin films. ZnS crystals typically exist in two phases: a cubic phase (zinc blende) that is stable at room temperature and a less dense hexagonal phase (wurtzite), which is stable at higher temperatures [37]. Annealing yields stress relief due to the readjust of Zn and S ions inside the ZnS lattice. Figure 2 shows the XRD pattern (20° ≤ 2θ ≤ 80°) for the as-deposited and annealed at 300 °C for one-hour ZnS thin films. It clearly gives a good indication of the polycrystalline structures of the obtained films.

Figure 2 shows a demanding peak at 34.8°, which represents the preferential orientation of H-ZnS (103) films. The less intense XRD peaks at 36.68° belong to the (H-105) lattice planes. A single diffraction peak was found in the XRD pattern as it is reflected from (200) direction and referred to the preferred orientation of cubic ZnS.

For the as-deposited ZnS films (Figure 2a), XRD pattern consists of Zn (OH)_2_ (011), C-ZnS (200), and H-ZnS (106). At the annealing temperature of 300 °C (Figure 2b), the intensity peak of Zn (OH)_2_ (011), H-ZnS (105) and (106), and C-ZnS (200) just started to be more intensive and the intensity peak of both C-ZnS (222) and H-ZnS (206) appeared.

The speeds of hydrolysis of thiourea are found to be increased as the temperature increases. This increase promotes a moderately high grouping of S^2−^ particle and generally low fixation OH- particle. Here and now, the ionic product of Zn^2+^ and S^2−^ particles surpasses the solubility product of ZnS, and then ZnS precipitation shapes in an arrangement. Unexpectedly, a low convergence of OH- particle is utilized to hydrolysis of thiourea and smothers the development of Zn (OH)_2_.

The lattice parameters a and c of the unit cell are given by the following equation [38,39],

(1) $$\frac{1}{d^{2}}=\frac{4}{3}\frac{h^{2}+\mathrm{hk}+k^{2}}{a^{2}}+\frac{l^{2}}{c^{2}}$$

where d is the interplanar spacing.

The volume of the unit cell for hexagonal crystals is given by:

(2) $$V=a^{2\;}c\;\sin\left(60\right)$$

The grain size (D) values; are evaluated by Scherrer equation [40],

(3) $$D=\frac{0.9\lambda }{\beta \cos\theta }$$

where λ is the wavelength of X-ray (1.5406 Å), β is (FWHM) and θ is the Bragg angle.

In addition, grain size (D) values; are evaluated by the Williamson–Hall method [41]. These values are shown in Table 1.

Dislocation density has been estimated by [42],

(4) $$\delta =\frac{1}{D^{2}}$$

where δ also refers to as the measure of the amount of defects in a crystal.

The number of crystallites per unit area (N) and the strain (ε) of the films were calculated using the following formulae [42]:

(5) $$N=\frac{t}{D^{3}}$$

(6) $$\varepsilon =\frac{\beta \cos\theta }{4}$$

where t is the thickness of the thin films.

The Scherrer formula considers only the effect of crystallite size on the XRD peak broadening. However, it does not show anything about the microstructures of the lattice. Williamson–Hall (W–H) is the easier and simplified method. In this method, physical line broadening of X-ray diffraction peak occurs due to the size and micro strain of the crystals.

Table 2 shows the influence of annealing temperature on the structure of the deposited hexagonal ZnS with a peak intensity of preferential orientation (103).

It is realized from Table 2 that since FWHM is reliant on the crystallite size and the lattice strain caused by the defect or dislocations, it decreases with the annealing as compared to the as-deposited thin film. The FWHM value decreased from 13.17° for as-deposited films to 8.99° for annealed films at 300 °C. In addition, as the grain size increases, lattice parameters (a and c), unit cell volume, dislocation density, the number of crystallites, and strain are all decreased with annealing at 300 °C. Such results have already been apparent by others [30,43]. This means that the crystallinity of films is extremely affected by the annealing temperature. It also is consistent with the highest peak intensity seen from the XRD patterns.

**Figure 3:**
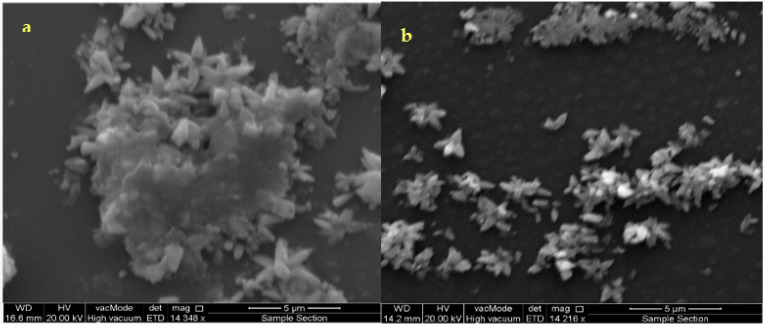
SEM microphotographs of the as-deposited ZnS thin film (**a**,**b**) annealed at 300 °C.

Figure 3a,b are SEM images of the as-deposited and annealed films at (300 °C) ZnS thin films.

(Figure 3a) shows the surfaces of the initial ZnS formation during the induction time, (i.e., at the start of deposition for as-deposited films). The as-deposited film has an inhomogeneous surface with some cracks and the reduced population of small grains on the surface of the substrate is detected. The surface morphologies of the grown ZnS films are shown in Figure 3b after annealing to 300 °C. It is progressively homogeneous and denser without any cracks. The grains are arbitrarily framed and medium in size. This gives the uniformity on the film surface to be observed. The growing process of the films is shown in Figure 3. It presents the formation of clusters on the surface, which is typical.

The clusters are formed by the aggregation of involved aqueous ions during the film deposition and growth. Using the same $\mathrm{Zn}{\left(\mathrm{OH}\right)}_{4}^{2-}{/\mathrm{HS}}^{-}$ concentration ratio, the desirable pH value achieved was found to be constant during the annealing process, i.e., a well-controlled chemical reaction maintained during deposition and growth of the ZnS films. Thus, it is suitable to adopt a clustering mechanism for the deposition and growth of the ZnS films under the chemical conditions imposed in this work. According to the estimated activation energy value at the initial ZnS film formation, there exists an intermediate adsorption process of the involved aqueous ions in the chemical solution/substrate surface, creating clusters and ZnS film during the induction time. During this growth process, the clusters of the involved ions may increase the adsorption progression resulting in heterogeneous catalysis in the chemical solution/substrate surface, which causes an increase of the thickness of the film in such a way where the film acts as a catalytic surface. Therefore, we believe that the growing process via clusters is controlled by the interaction of zinc hydroxide ions with the bisulfite ions in the frontier between the chemical solution/ZnS film and the substrate surface. Furthermore, it can be seen from Figure 3 that the deposited films show a flower-like structure. A better quality of the samples was obtained at the annealing temperature of 300 °C, though the oxidation process is blocked when the layer is annealed under the vacuum. The annealing process gives a chance to rearrange the atoms that make larger grain sizes and well-ordered primitive crystalline cells.

From Table 1 and correspondingly, with the analysis of the results XRD above (Figure 2), since the likelihood of oxidation increments at higher annealing temperature, it is essential to keep the annealing temperature in such ranges, where the crystallinity and transmittance are improved with no sorts of outer impacts.

**Figure 4:**
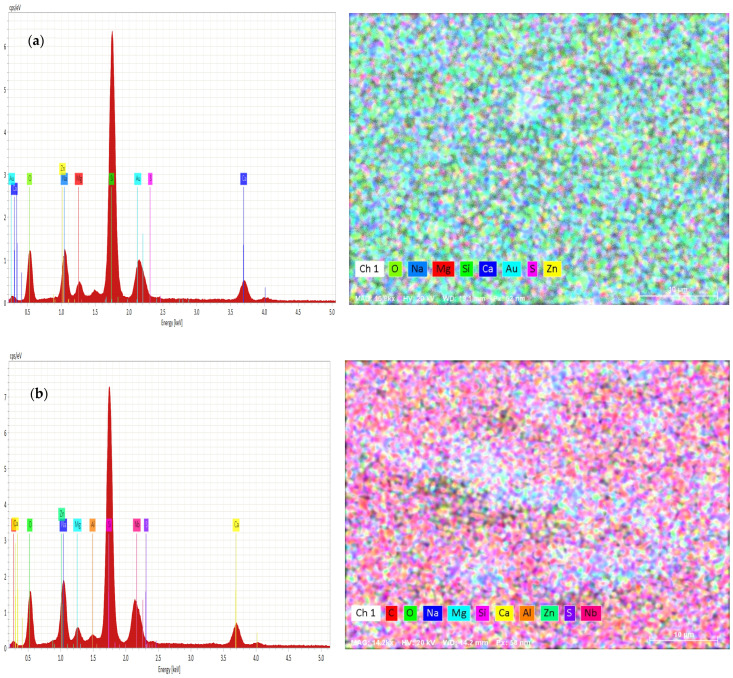
(**a**) EDAX of as-deposited ZnS thin films and (**b**) EDAX of ZnS thin films annealed at 300 °C.

The variation of chemical compositions of Zn and S was analyzed and evaluated by EDAX, as shown in Figure 4a,b.

From Figure 4a,b, the EDAX analysis confirms the presence of zinc and sulfur in the obtained films. The annealed sample had more Zn content than S. In addition, oxygen contents were increased with the annealing temperature of 300 °C. The annealing of the samples caused a decrease in the concentration of S atoms. An excess of Zn may be due to the amount of Zn (OH)_2_ or ZnO originating from the alkaline reaction solution.

### 3.2. Optical Analysis

Optical characteristics were examined by measuring the transmittance and absorbance at the range of 310–1100 nm for the as-deposited and annealed ZnS films.

**Figure 5:**
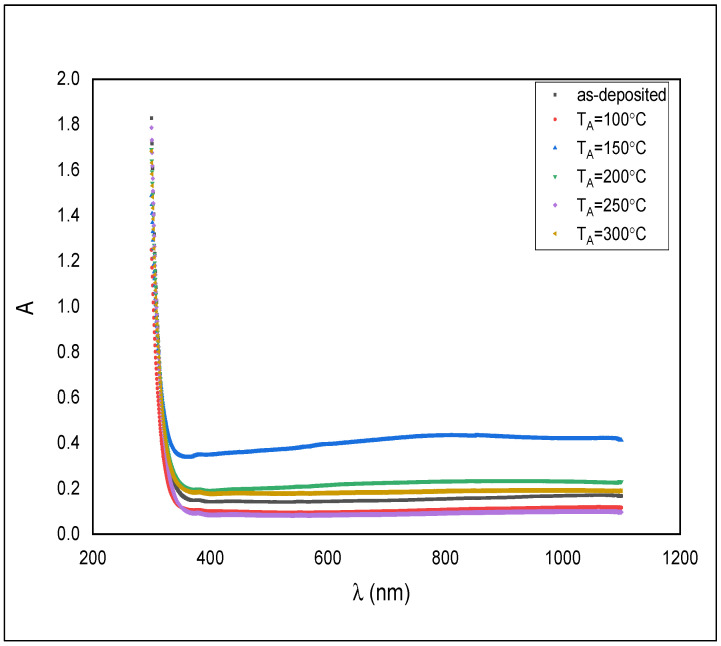
Absorbance spectra for the as-deposited and annealed ZnS films.

Figure 5 shows the absorption spectra of as-deposited ZnS thin films and annealed at temperatures of 100, 150, 200, 250, and 300 °C.

All samples displayed higher absorbance in the UV region rather than near the IR region. The absorbance is just somewhat in the visible region. That makes the material beneficial as a windscreen covering and driving mirror to forestall the impact of striking light into a driver’s eyes from an approaching vehicle and following vehicle.

**Figure 6:**
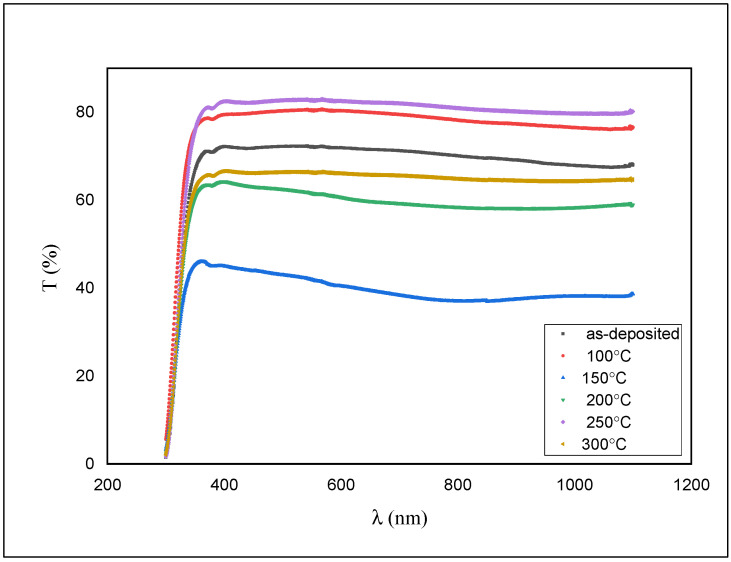
Transmission spectra for as-deposited and annealed ZnS films.

The transmittance measurements of the films were done using UV/VIS spectrophotometer and are shown in Figure 6.

As shown in Figure 6, the transmission is around 76% for the as-deposited film and diminished to ~50% with an expansion in the annealing temperature.

**Figure 7:**
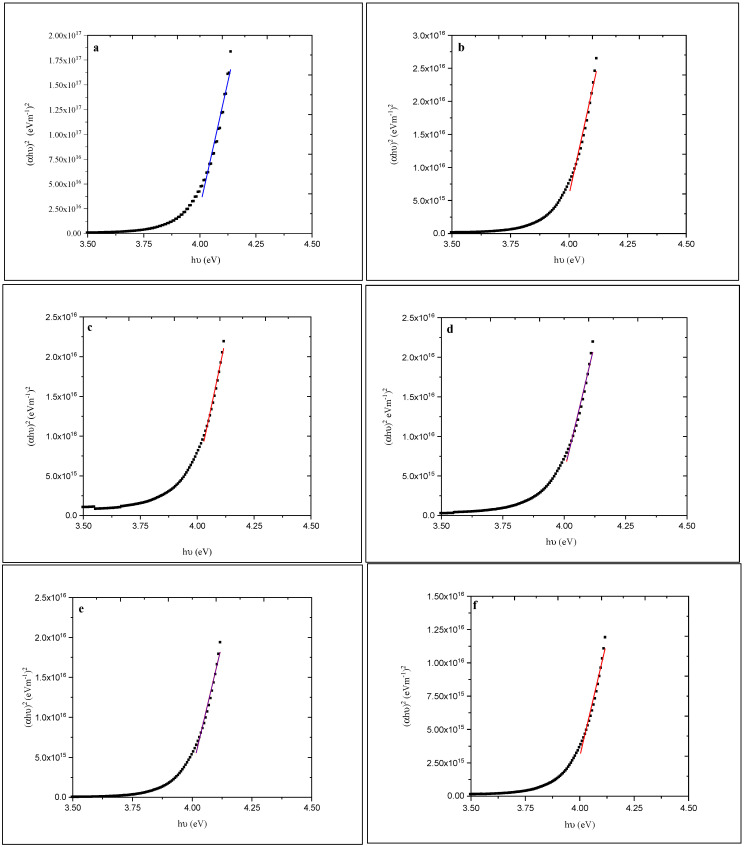
Plots of (αhυ)^2^ versus hυ for (**a**)-as-deposited and annealed (at (**b**) 100, (**c**) 150, (**d**) 200, (**e**) 250, and (**f**) 300 °C) ZnS films.

To calculate the optical energy gap, a plot of (αhν)^2^ versus hν (where α is the optical absorption coefficient and hν is the energy of the incident photon) was made as shown in Figure 7.

The energy gap (Eg) was determined using the relationship [44]:

(7) $$\alpha h\upsilon =K{\left(h\upsilon -\mathrm{Eg}\right)}^{1/2}$$

where K is a constant.

**Figure 8:**
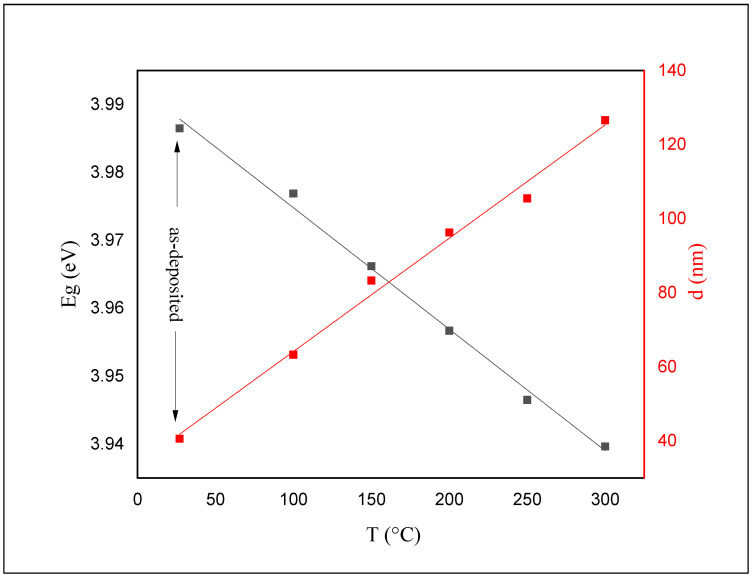
Energy gap and thickness for as-deposited and annealed ZnS thin films as a function of annealing temperature.

The calculated Eg and thickness values for both as-deposited and annealed films are shown in Figure 8.

The value of Eg for the as-deposited ZnS films was calculated to be 3.98 eV. This value has been reduced to 3.93 eV after annealing. The color of the as-deposited films was white before and after annealing. The absorption edge shifts gradually toward longer wavelengths and shrinks the bandgap (see Figure 6 and Figure 7) as the annealing temperature increases.

## 4. Conclusions

ZnS thin films were prepared using the chemical bath deposition technique. The structural properties for as-deposited and annealed films at 300 °C were examined. In addition, the optical properties for as-deposited and annealed films at 100, 150, 200, 250, and 300 °C were also investigated. XRD analysis shows the polycrystalline structures of the grown films. It was found that the FWHM is dependent on the crystallite size and the lattice strain caused by the defect or dislocations. The FWHM had been reduced with annealing. Correspondingly, SEM images of the annealed ZnS films were shown to be moderately denser and more homogeneous without any cracks. However, relatively small-sized grains were randomly formed, and the as-deposited film had seen to have an inhomogeneous surface with some cracks. Other than that, the annealed samples were observed to have more Zn content than S. The Zn/S proportion had been diminished gradually with annealing. The optical characterization reveals that the transmittance was around 76% of the as-deposited film and decreased to ~50% with the increasing of annealing temperature. The bandgap energy of the as-deposited film was 3.98 eV and was found to be decreased to 3.93 eV after annealing. In addition, the white color of the as-deposited thin films did not change after annealing.

## Figures and Tables

**Table 1:** Grain size (D) values of ZnS with a peak intensity of (103) plane using Scherrer equation and the Williamson–Hall method.

Annealing Temperature (°C)	D (Å)	D (Å)
	Scherrer equation	Williamson–Hall method
as-deposited	352.52	322.97
300	528.95	503.06

**Table 2:** X-ray analysis of hexagonal ZnS with a peak intensity of (103) plane.

Annealing Temperature (°C)	2θ (Degree)	β	c (Å)	a (Å)	D (Å)	d (Å)	V (Å)^3^	δ (Lines/m^2^) ×10^−6^	N ×10^14^	ε ×10^−4^
as-deposited	34.85	0.23	5.143	2.96	352.52	2.573	246.38	8.046	9.27	9
300	34.82	0.157	5.148	2.972	528.95	2.576	247.08	3.574	8.55	6

## Data Availability

Not applicable.
